# The Institute of Physical Medicine and Rehabilitation, Hospital das Clínicas University of São Paulo School of Medicine comprehensive rehabilitation program for elderly people with knee osteoarthritis

**DOI:** 10.3389/fmed.2022.1029140

**Published:** 2022-11-09

**Authors:** Marta Imamura, Gilson T. Shinzato, André T. Sugawara, Sabrina Saemy Tome Uchiyama, Denise Matheus, Marcel Simis, Denise Vianna Machado Ayres, Artur C. A. dos Santos, Tatiane Assone, Vinícius Delgado Ramos, Felipe Fregni, Linamara R. Battistella

**Affiliations:** ^1^Instituto de Medicina Física e Reabilitação, Hospital das Clínicas da Universidade de São Paulo, São Paulo, Brazil; ^2^Departamento de Medicina Legal, Bioética, Medicina do Trabalho e Medicina Física e Reabilitação, Faculdade de Medicina da Universidade de São Paulo, São Paulo, Brazil; ^3^Faculdade de Medicina da Universidade de São Paulo, São Paulo, Brazil; ^4^Department of Physical Medicine and Rehabilitation, Massachusetts General Hospital, Boston, MA, United States; ^5^Harvard Medical School, Boston, MA, United States

**Keywords:** knee osteoarthritis (KOA), focal extracorporeal shockwaves, radial pressure waves, neuromuscular electrical stimulation, Lidocaine paraspinous block, rehabilitation

## Abstract

**Background:**

Knee osteoarthritis (OA) is a leading cause of disability in the elderly population. Chronic disabling pain is associated with maladaptive neuroplastic changes in brain networks, commonly associated with central sensitization. The main clinical features of nociplastic pain conditions include combined peripheral and central sensitization, and it is crucial to recognize this type of pain, as it responds to different therapies than nociceptive and neuropathic pain.

**Objective:**

To report the effect of the Institute of Physical Medicine and Rehabilitation (IMREA) comprehensive rehabilitation program to reduce pain and to improve functioning in elderly people with knee OA, under the DEFINE cohort.

**Methods:**

This is a retrospective observational cohort of 96 patients with knee OA, recruited from October 2018 to December 2019. All patients were evaluated by a trained multidisciplinary team using the Kellgren Lawrence classification, bilateral knee ultrasonography, the visual analog scale (VAS), the Western Ontario and McMaster Universities Arthritis Index (WOMAC) pain, rigidity and difficulty scores, the Timed Up and Go Test (TUG), 10-m and 6-min walking test (10 and 6 MWT), Berg Balance Scale, isokinetic dynamometry for knee extension and flexion strength, and pain pressure thresholds. The rehabilitation program included paraspinous lidocaine blocks, focal extracorporeal shockwaves combined with radial pressure waves and functional electrical stimulation according to individual needs. The baseline was compred with the treatment results with a paired *t*-test.

**Results:**

The study sample is composed of 96 participants, mostly females (*n* = 81, 84.38%), with bilateral osteoarthritis (*n* = 91, 94.79%), and a mean age of 68.89 (SD 9.73) years. Functional improvement was observed in TUG (*p* = 0.019), 6-mwt (*p* = 0.033), right knee flexion strength (*p* < 0.0001), WOMAC rigidity and difficulty domains (*p* < 0.0001). Pain was reduced from baseline as measured by WOMAC pain domain (*p* < 0.0001), VAS for both knees (*p* < 0.0001), and SF-36 pain domain (*p* < 0.0001). Pressure pain threshold was modified above the patella (*p* = 0.005 and *p* = 0.002 for right and left knees, respectively), at the patellar tendons (*p* = 0.015 and *p* = 0.010 for right and left patellar tendons, respectively), left S2 dermatome (*p* = 0.017), and L1-L2 (*p* = 0.008).

**Conclusions:**

The IMREA comprehensive rehabilitation program improved functioning and reduced disabling pain in elderly people with knee OA. We highlight the relevance and discuss the implementation of our intervention protocol. Although this is an open cohort study, it is important to note the significant improvement with this clinical protocol.

## Introduction

Knee osteoarthritis (OA) is a leading cause of disability and source of societal cost in older adults, and is the most common form of arthritis worldwide (1). Previous studies have reported that age, sex, obesity, genetics, injury, and low educational levels was related with higher prevalence of OA (2).

Several non-pharmacological and non-surgical interventions are recommended as the first line of treatment for OA. These measures relieve pain while maximizing functioning and quality of life, and reducing adverse effects from drugs and invasive interventions (3).

However, comorbid conditions and concurrent interventions may influence the outcome of rehabilitation interventions (3–5). Unfortunately, the magnitude of the effects of most interventions are low and the number of total knee replacements is exponentially increasing (6). Therefore, better understanding of the mechanisms involved in pain generation and propagation is important to improve outcomes in these patients.

Chronic disabling pain is associated with maladaptive neuroplastic changes in brain networks, commonly associated with central sensitization (7, 8). This phenomenon is recently recognized and classified as nociplastic pain (9). It is defined as pain that arises from altered nociception despite no clear evidence of actual or threatened tissue damage causing the activation of peripheral nociceptors or evidence for disease or lesion of the somatosensory system causing the pain (10). Nociplastic pain derives from augmented pain processing and altered pain modulation in the central nervous system and should be considered in any patient with chronic pain (11).

Main clinical features of nociplastic pain conditions include combined peripheral and central sensitization, spinal cord reorganization, hyper-responsiveness to painful and non-painful sensory stimuli, associated with fatigue, sleep and cognitive disturbances, anxiety and depression mood (10, 11). It is crucial to recognize this type of pain, as it responds to different therapies than nociceptive and neuropathic pain. In this way, the objective of the present manuscript was to report the IMREA clinical and therapeutic measures that improved the pain and functional evaluation in elderly with knee osteoarthritis, under the DEFINE study (4).

## Methods

### Cohort characteristics

Retrospective open cohort is composed of patients with knee osteoarthritis. All subjects were admitted to the conventional outpatient rehabilitation program of the Institute of Physical Medicine and Rehabilitation Hospital das Clínicas (HCFMUSP) University of São Paulo School of Medicine (IMREA).

### Participants and study design

**Figure 1:**
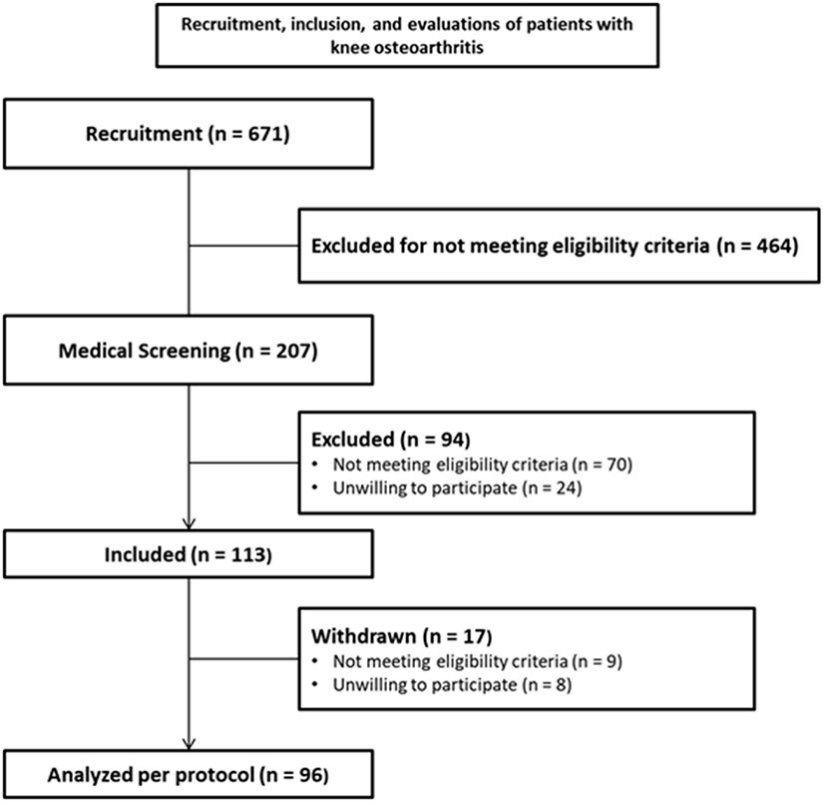
Description of patients with knee osteoarthritis (OA) were recruited and included in the present study.

For this study, 113 patients with knee osteoarthritis (OA) were recruited from October 2018 to December 2019, the sample was selected by convenience. The sample size of 100 patients was determined for the longitudinal cohort (DEFINE Study) (4). The Figure 1 describes the process of volunteer's recruitment, screening, inclusions, and clinical evaluations. All patients who agreed to participate in the study and signed the informed consent form underwent a series of assessments at two time points: before and after the IMREA rehabilitation program. The personalized and individualized program systematically assessed for clinical findings of sensitization, presence of periarticular lesions and other sources of pain.

### Inclusion criteria

Participants of both sexes were included in the study if they were older than 50 years, have confirmed clinical stability verified by medical evaluation (12), have signed the informed consent form, and if they fulfilled the eligibility criteria for the IMREA rehabilitation program and confirmed knee OA in plain radiography.

### Exclusion criteria

Concomitant manifestations in other joints, secondary knee osteoarthritis, clinical instability, and social conditions.

### Clinical and functional evaluation of knee osteoarthritis

#### Clinical evaluation

After a full patient's history and physical examination, knee OA was confirmed using RX and classified in the Kellgren Lawrence criteria. Sociodemographic data included age, sex, and body mass index. We reported the main medications used by recruited patients when included in the study. The medication regimen was not modified during the interventions. Simple analgesics were used by 32.3% of the patients, non-steroidal anti-inflammatory drugs by 9.4%, muscle relaxants by 13.5% and weak opioids (tramadol or codeine) by 6.3%.

#### Functional clinical assessment

Pain intensity was obtained by a visual analog scale (VAS) (13, 14) per right or left sides. WOMAC pain score per person, SF-36 pain domain. Bilateral pain pressure thresholds were measured twice at 1 inch above the patella, over the patellar tendon, at the adductor longus muscles, at the S2 dermatome and at the L1-L2 supraspinous ligaments (7). Mean value of the two measurements were analyzed for the right and the left sides.

Knee functioning was evaluated using the WOMAC scores (WOMAC rigidity and WOMAC difficulty) per person (15, 16), Timed Up and Go Test (TUG) (17), the 10-m (10 MWT) (18) and the 6-min walking test (6 MWT) (19), Berg Balance Scale (20), bilateral isokinetic dynamometry for knee extension and flexion strength (21).

##### Knee radiographic evaluation

We assessed three incidences: anteroposterior (AP) with load, lateral and axial patella, using a Luminus RF X-ray equipment (Siemens, Germany). Classification of KOA severity was performed using the Kellgren-Lawrence scores. The same radiologist analyzed all X-ray exams.

##### Kellgren Lawrence radiographic classification of OA

Zero (without osteoarthritis) to four (large osteophyte, marked narrowing of the joint space, severe sclerosis, and definite deformity of bony extremities).

##### Ultrasonographic evaluation

Knee USG evaluations were performed using Siemens Sonoline G40 USG equipment with multi-frequency linear transducers of 5–13 MHz (Siemens, Germany). Images were obtained in the axial plane (transversal) to assess the patellar region, followed by the longitudinal plane, the enthesis with insertion of the quadriceps tendon, the patellar tendon (proximal and distal), as well as the medial and lateral collateral ligaments, the topography of the iliotibial tract and the components of the pes anserinus bursa. Subsequently, patients were placed in ventral decubitus where the axial and longitudinal planes allowed the visualization of the popliteal fossa. In this view, we were able to assess the presence of effusion as well as of a popliteal cyst, also known as Baker's cyst.

#### Outpatient rehabilitation program

**Figure 2:**
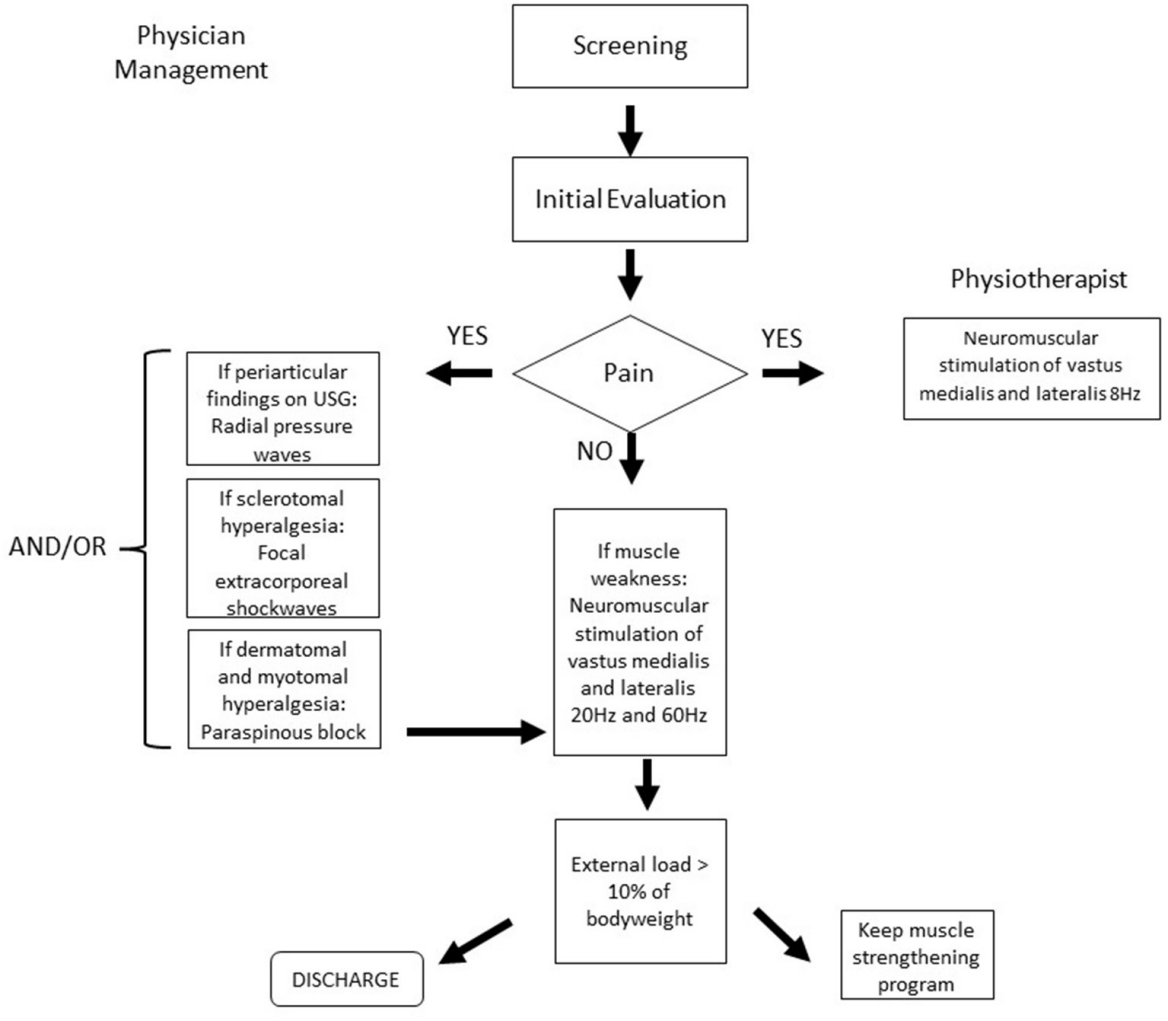
Intervention decision tree. USG, ultrasound imaging.

The rehabilitation program provided at the Institute of Physical Medicine and Rehabilitation (IMREA) consisted of weekly visits, administered once or twice a week, according to the individualized and personalized rehabilitation needs based on the results of the multidisciplinary evaluations. Pain was managed according to clinical and functional findings, in a hierarchical scheme (Figure 2).

##### Focal shock waves

The treatment was delivered through an electromagnetic generator Duolith Ultra SD1 (STORZ Medical, Tagerwillen, Switzerland). The focal shockwave was set to reach between 4.0 and 6.0 cm depth (5.0 cm center of focus), with energy flux density fixed at 0.12 mJ/mm^2^ for all regions and patients, and deliver up to 2,000 pulses per treated segment.

This original approach, are applied in IMREA since 2017, and it allowed the development of a new method of intraosseous algometry, diverging from the current methods that register the painful pressure threshold at soft tissues. A verbal pain score was registered at the beginning of the stimulus and at the end of the shock waves train of 2,000 pulses for each segment treated. When the level zero of pain was achieved before reaching these 2,000 pulses, the application was interrupted and the number of pulses was registered (22).

The sessions were conducted once a week for five consecutive weeks or as needed. We have systematically searched for areas of hyperalgesia at the femoral greater trochanter, and the three knee compartments: medial, patellofemoral and lateral, emphasizing specific areas of main clinical relevance, medial tibial plateau, trans patellar, femoral trochlea and the medial posterior area of the tibial plateau and femoral condyle. Focal shock wave therapy was applied in 70.8% of the participants (*n* = 68) who received an average of 3.86 sessions (**Table 2**).

##### Radial pressure waves

We applied 2,000 radial pressure waves using a pneumatic generator, Swiss DolorClast^®^ (EMS Electro Medical Systems, Nyon, Switzerland). We used the Power^®^ handpiece, at a frequency of 15–20 Hz, and with individual acoustic pressure of 2.5–4.0 bar, selected according to the patient's pain tolerance. Radial pressure waves were applied weekly, for three consecutive weeks over areas previously identified by the ultrasonographic evaluations including the pes anserinus bursitis, patella tendinitis, quadriceps tendinitis and at the tensor fascia lata. We also included the application over the medial intra articular line. The radial pressure waves therapy was applied in 47.91% of the participants (*n* = 46) who received a mean of 2.13 sessions (**Table 2**).

##### Lidocaine paraspinous block

When dermatomal and subcutaneous sensitization at a metameric distribution was clinically detected by the pinch and roll maneuver (7), even after the previous interventions, we combined the Lidocaine paraspinous block of the affected spinal segmental level(s) (23). We used lidocaine at 1% concentration, without epinephrine, using 25–27 G, 3.7 cm length disposable needles. The paraspinous block was applied into the space between the spinous process and the spinalis muscle, performed by experienced physicians, weekly, as needed. The most commonly affected spinal level in these KOA patients was L3. Lidocaine paraspinous block was performed in 45.83% of the participants (*n* = 44) who received an average of 2 blocks (**Table 2**).

### Neuromuscular electrical stimulation

The contraction of the quadriceps *vastus medialis* and *vastus lateralis* muscles was induced by neuromuscular electrical stimulation using FESMED IV equipment (CARCI, São Paulo, Brazil), which, in Functional Electrical Stimulation (FES) mode, produces a biphasic, square and pulsed wave. The active electrode was positioned over the *vastus medialis* and *vastus lateralis* muscles and the neutral electrode in the proximal anterior region of the thigh. The stimulation began after mapping and placing the electrodes on specific sites to obtain the best muscle contraction. The following stimulation parameters were used: ascent time: 0 s; descent time: 1.0 s; duration of stimulation time: 5 s; duration of rest time: 10 s; pulse width: 500 μs (microseconds); stimulation frequencies: 8, 20, and 60 Hz; stimulus intensity (milliamperes): according to the patient's tolerance and progressive external load. The frequency of 8 Hz was used for hyperalgesia of the dermatome (24), and 20 and 60 Hz for muscle contraction (25, 26).

The patient was positioned in the supine position, with a cushion below the knee in 30° flexion. When the knee was fully extended during electrical stimulation an external load was gradually applied on the distal third of the leg until it reached the functional goal of 10% of body weight. The patient achieving complete electrically induced knee extension for 30 min, without active movement after reaching the functional goal, the patient was discharged from the NMES program. The session lasted 45 min and was conducted twice a week. The neuromuscular electrical stimulation therapy was applied in 100% of the participants (*n* = 96) who received an average of 13.64 sessions (**Table 2**).

### Data quality control

Data collection was performed by the main researcher with the help of residents and doctors of the knee osteoarthritis outpatient clinic of the Institute of Physical Medicine and Rehabilitation, Hospital das Clínicas (HCFMUSP). The data entry was performed by two people from the administrative sector of the research team, and subsequently these data were checked by the main researcher. The results were tabulated in the electronic database using Excel^®^ (Microsoft, CA).

### Ethical issues

Participants with a clinical and radiological diagnosis of knee osteoarthritis were invited to participate in the study and included after signing the informed consent form previously approved by the Hospital das Clínicas da Faculdade de Medicina da Universidade de São Paulo Ethics Committee for Research Protocol Analysis CAAE: 86832518.7.0000.0068.

### Statistical analyzes

The continuous variables were described as means and standard deviation and categorical data was described as absolute and relative frequencies. Changes from baseline in pain and functional assessments were tested with paired *t*-test given the sample size and the possibility of applying the central limit theorem (27). The function outcome considered eight assessments (TUG, 6 MWT, 10 MWT, knee extension strength, knee flexion strength, BBS, WOMAC rigidity, and WOMAC difficulty) and pain with four (WOMAC pain domain, VAS for knee pain intensity, SF-36 pain domain, and lower limb PPT), statistical significance was considered for the alpha level of 0.05. All the statistical tests were conducted with the statistical pack Stata 14.

## Results

As demonstrated in Figure 1, the study included 113 participants, however, after inclusion and during the interventions, 17 patients were withdrawn from the study. Nine participants presented some discontinuation criteria. Eight patients dropped out of the study: four withdrew their informed consent and four others to treat other health conditions they discovered after entering the study (hemochromatosis, polyneuropathy, spine stenosis, and osteopathy). Therefore, the analysis of treatment outcomes was conducted with the remaining 96 patients.

The analyzed sample were mostly composed of females (*n* = 81, 84.38%), with bilateral osteoarthritis (*n* = 91, 94.79%), and mean age of 68.89 (SD 9.73) years. The baseline characteristics of the participants are shown in Table 1.

**Table 1:** Patients characteristics at baseline.

**Baseline (*N* = 96)**	**Mean (SD) or *N* (%)**	**Min–Max**	**95% CI**
Females/Males, *N* (%)	81/15 (84.38%/15.63%)	-	-
Age (years)	68.89 (9.73)	50.76–99.91	66.91–70.86
Body mass index	31.93 (5.34)	19.91–48.68	30.85–33.02
Time since diagnosis (months)	99.33 (101.87)	3–492	76.69–119.97
Bilateral Osteoarthritis, *N* (%)	91 (94.79%)	-	-
Total knee replacement, *N* (%)	5 (5.21%)	-	-
K&L right/left, *N* (%)			
Level 0	0/1 (0%; 1.08%)		
Level I	26/29 (27.66%; 31.18%)	-	-
Level II	25/24 (26.60; 25.81)	-	-
Level III	14/16 (14.89; 17.20)	-	-
Level IV	29/23 (30.85; 24.73)	-	-
USG findings right/left, *N* (%)			
Effusion	73 /70 (77.66%; 75.27%)	-	-
Pes anserinus bursitis	43/40 (45.74%; 43.01%)	-	-
Iliotibial band tendinitis	31/18 (32.98%; 19.35%)	-	-
Enthesopathy of quadriceps tendon	29/29 (30.85%; 31.18%)	-	-
Popliteal cyst	28 /27 (29.79%; 29.03%)	-	-
Patellar tendinitis	15/13 (15.96%; 13.98%)	-	-
Timed Up and Go (seconds)	15.76 (7.94)	7.78–63.62	14.15 - 17.38
6 min-walk test (m)	311.06 (107.54)	55.7–613.6	289.16–332.97
10 m walk test (s)	11.94 (7.19)	5.94–48.03	10.47–13.40
Berg balance scale	48.24 (8.42)	13–56	46.53–49.96
Right knee extension strength (Nm)	61.82 (30.98)	11–156	55.37–68.28
Left knee extension strength (Nm)	62.56 (29.33)	9–175	56.45–68.67
Right knee flexion strength (Nm)	44.78 (19.39)	11–95	40.74–48.82
Left knee flexion strength (Nm)	46.74 (19.99)	8–137	42.57–50.90
WOMAC rigidity	4.55 (2.03)	0–8	4.13–4.96
WOMAC difficulty	35.62 (14.23)	8–61	32.73–38.59
WOMAC pain	6.99 (4.22)	1–19	9.90–11.54
Visual Analog Scale, right knee	5.65 (2.87)	0–10	5.07–6.23
Visual Analog Scale, left knee	5.34 (2.85)	0–10	4.76–5.91
SF36-pain	40.26 (22.92)	0–100	35.59–44.93
PPT 1 inch above right patella	5.09 (3.03)	1.65–16.75	4.48–5.70
PPT 1 inch above left patella	5.18 (2.87)	1.3–16.25	4.60–5.77
PPT right patellar tendon	5.92 (3.21)	1.55–16.25	5.27–6.58
PPT left patellar tendon	5.88 (3.13)	1.65–15.85	5.25–6.52
PPT right adductor longus	3.06 (1.39)	1.15–8.35	2.78–3.34
PPT left adductor longus	2.92 (1.43)	1.15–8.0	2.63–3.21
PPT right S2 dermatome	2.70 (1.36)	0.75–9.32	2.42–2.97
PPT left S2 dermatome	2.58 (1.36)	1–8.25	2.29–2.84
PPT L1-L2	5.74 (3.06)	1.75–15.15	5.12–6.36

N, number; %; percentage; SD, standard deviation; Min, minimum; Max, maximum; CI, confidence interval; K&L, Kellgren & Lawrance scale; USG, ultrasonography; Nm, newton meters; WOMAC, Western Ontario and McMaster Universities Osteoarthritis Index; SF-36, 36-Item Short Form Survey; PPT, pain pressure threshold.

According to the Shapiro-Wilk tests, most variables are non-parametric, however we applied the central limit theorem given the sample size. Therefore, the continuous variables were described as means and standard deviations, and the primary analyses were conducted with the paired *t*-test.

The distribution of all interventions and the number of patients involved is presented in Table 2. Neuromuscular electrical stimulation was the only intervention that did not overlap the other treatments in 10 patients, whereas all the other techniques were combined.

**Table 2:** Treatment distribution.

**Interventions**	**Patients treated, *N* (%)**	**Min-max sessions/infusions**	**Mean sessions/infusions (SD)**	**Single intervention, *n* (%)**
Lidocaine paraspinous block	44 (45.83%)	1–7	2.0 (1.38)	0
Focal SWT	68 (70.8%)	1–6	3.87 (1.53)	0
Radial PWT	46 (47.91%)	1–7	2.13 (1.34)	0
NMES	96 (100%)	1–51	13.64 (11.43)	10 (8.85%)
NMES 8Hz	46 (47.92%)	-	-	-
NMES 20Hz	95 (98.96%)	-	-	-
NMES 60Hz	45 (46.88%)	-	-	-

N, number; %; percentage; Min, minimum; Max, maximum; SD, standard deviation; SWT, shockwave therapy; PWT, pressure wave therapy; NMES, neuromuscular electrical stimulation; Hz, Hertz.

Also, 100% of the participants received at least one NMES session, therefore, all interventions were combined with NMES. The median time of the treatment was 5.93 weeks (IQR 2.43–13), considering either the NMES as a single intervention or the combination of interventions.

Table 3 presents the treatment overlap and the number of patients in each combination. In Table 3, the four margins show the interventions, and all the possible combinations are the junctions of the margins. A hyphen was placed at the repeated combinations to avoid misinterpretation of the data.

**Table 3:** Treatment overlap.

	**NMES** ***N*** **(%)**			**Radial pressure Waves** ***N*** **(%)**	
Paraspinous block	14 (12.40%)	-	-	-	NMES
Radial PWT	10 (8.85%)	11 (9.73%)	-	-	
Focal SWT	15 (13.27%)	11 (9.73%)	28 (24.78%)	14 (12.39%)	
	Fischer	Radial PWT	Focal SWT	Paraspinous block	

N, number; %; percentage; NMES, Neuromuscular Electrical Stimulation; SWT, shockwave therapy; PWT, Pressure Wave Therapy.

The paired *t*-tests for function and pain improved after the interventions are described on Table 4. We have not observed any side effects or harm in any of the patients during the treatment program.

**Table 4:** Treatment outcomes.

**Outcomes (*N* = 96)**	**Baseline mean (±SD)**	**After treatment mean (±SD)**	***p*-value**
Timed Up and Go (Seconds)	15.76 (7.94)	14.80 (7.39)	**0.019**
6-min Walk Test, (meters)	313.72 (104.95)	334.75 (121.92)	**0.033**
10-meter Walk Test (seconds)	11.94 (7.19)	11.19 (6.61)	0.097
Right knee extension strength (Nm)	62.71 (31.76)	64.35 (30.18)	0.219
Left knee extension strength (Nm)	64.35 (29.38)	64.19 (29.09)	0.897
Right knee flexion strength (Nm)	45.32 (19.82)	48.69 (19.41)	**< 0.0001**
Left knee flexion strength (Nm)	47.94 (20.09)	49.25 (20.22)	0.201
Berg Balance Scale	48.24 (8.42)	49.45 (6.73)	0.064
WOMAC rigidity	4.55 (2.03)	3.28 (2.16)	**< 0.0001**
WOMAC difficulty	35.96 (14.31)	25.21 (14.49)	**< 0.0001**
WOMAC pain	10.73 (4.03)	7.02 (4.23)	**< 0.0001**
Visual Analogue Scale right knee	5.65 (2.87)	2.66 (2.69)	**< 0.0001**
Visual Analogue Scale left knee	5.34 (2.85)	2.12 (2.23)	**< 0.0001**
SF36-pain	40.22 (22.44)	54.48 (21.65)	**< 0.0001**
SF36-pain, male subgroup	51.83 (28.79)	53.83 (21.58)	0.621
SF36-pain, female subgroup	37.95 (20.49)	54.61 (21.80)	**< 0.0001**
Pain Pressure Threshold (kg/cm^2^)			
One inch above right patella	5.09 (3.03)	5.99 (3.34)	**0.005**
One inch above left patella	5.18 (2.87)	6.17 (3.28)	**0.002**
Right patellar tendon	5.92 (3.21)	6.73 (3.12)	**0.015**
Left patellar tendon	5.88 (3.13)	6.72 (2.96)	**0.010**
Right adductor Longus	3.06 (1.39)	3.29 (1.62)	0.137
Left adductor Longus	2.92 (1.43)	3.10 (1.59)	0.294
Right S2 dermatome	2.69 (1.36)	2.93 (1.57)	0.095
Left S2 dermatome	2.57 (1.36)	2.98 (1.74)	**0.017**
L1-L2	5.74 (3.05)	6.56 (3.27)	**0.008**

SD, Standard deviation; *p*-value, Paired *t*-test; N, number of patients; Nm, newton meters; WOMAC, Western Ontario and McMaster Universities Osteoarthritis Index; SF-36, 36-Item Short Form Survey; S2, sacrum; L1 and L2, lumbar.

Bold significant values (*p* < 0.05).

## Discussion

The IMREA comprehensive rehabilitation program for elderly people with knee osteoarthritis reduces pain and improves their functioning status in a short period of time of 5.93 weeks (IQR 2.43–13). The rehabilitation program significantly improved VAS scores, WOMAC pain, rigidity and difficulty subscales and the SF-36 pain subscale. Other parameters including the TUG, 6-min walking test, flexor isokinetic muscle strength also improved after the intervention. The present report presented the rehabilitation program in detail, as it employed a combination of strategies to reduce sensitization at the peripheral and spinal levels, as well as the vastus medialis and vastus lateralis muscle capacity. The IMREA approach rationale was patient centered, strategizing to target individual needs. As a cohort study, the DEFINE primary goal was not to test the efficacy of individual interventions, included in the rehabilitation program.

Interestingly, IMREA program focused on previously described physical examination findings of dermatomal, myotomal and sclerotomal hyperalgesia (7). We employed an approach to clinically identify nervous system hyperalgesia in patients with disabling knee OA pain, using pressure pain threshold measures in various anatomical regions (7). The better understanding of the mechanisms involved in how knee OA pain is generated, and how the sensory information is processed from peripheral receptors to cerebral cortex (1), provided useful insights that lead to the clinical benefits we were able to capture. Initially, hypersensitivity is found at the site of damage; however, when the disease process is not controlled, such as in patients with OA and refractory pain, the central nervous system undergoes plastic changes that are responsible for sustaining chronic pain. It then becomes independent from the peripheral pathologic process. In fact, we identified lower PPT values in various anatomical structures further away from the knee joint. We used focal extracorporeal shockwaves to manage pain deriving from the knee joint. The present protocol also used ultrasonography to identify peripheral soft tissue changes including patella tendinitis, pes anserinus bursitis among others (28). For these periarticular soft tissue abnormal findings, we used radial pressure wave therapy. In cases of decreased pressure pain thresholds within a metameric pattern, we indicated a paraspinous block at the involved spinal segments. For patients presenting muscle weakness, electrically induced muscle strengthening was used. The number of sessions for each individual intervention varied among included patients up to discharge criteria.

The IMREA rehabilitation program based on data from previous findings that centrally induced neuroplastic changes measured by a decreased pressure pain threshold over superficial and deep structures may also occur in areas distant from the knee area. For example, spinal segmental sensitization is a hyperactive state of the spinal cord caused by repeated stimulation of nociceptive receptors from impulses sent by sensitized damaged tissue to the dorsal horn neurons (central nervous system sensitization). The mechanisms of spinal segmental sensitization include neuron hypertrophy and up-regulation of excitatory neurons and of pro hyperalgesic peptides, and neurotransmitters at the dorsal horn of the spinal cord. This result in a mismatch of inflammation and pain, as pain does not indicate worsening of inflammation and vice versa.

This phenomenon is recently recognized and classified as nociplastic pain (9). It is defined as pain that arises from altered nociception despite no clear evidence of current or potential tissue damage causing the activation of peripheral nociceptors or evidence for disease or lesion of the somatosensory system causing the pain (10). Nociplastic pain derives from augmented pain processing and altered pain modulation in the central nervous system and should be considered in any patient with chronic pain. It is a phenotypic expression of multifactorial processes originating from different inputs, both as a response to a peripheral nociceptive or neuropathic trigger and reduced pain inhibitory mechanisms (11). Main clinical features of nociplastic pain conditions include combined peripheral and central sensitization, spinal cord reorganization, hyper-responsiveness to painful and non-painful sensory stimuli, associated with fatigue, sleep and cognitive disturbances, hypersensitivity to environmental stimuli, anxiety and depression mood. It is crucial to recognize this type of pain, as it responds to different therapies than nociceptive and neuropathic pain. Indeed, we have shown in previous analyses of this cohort that higher alpha and beta power on electroencephalogram (EEG) appears to be a compensatory mechanism associated with severe joint degeneration and reduced motor function (29). Similarly, higher intracortical inhibition as indexed by transcranial magnetic stimulation (TMS) intracortical inhibition are associated with younger age, greater cartilage degeneration (as seen by radiographic severity), less pain in WOMAC scale in OA subjects (30). These results underscore the role of central nervous dysfunction and neuroplastic changes in knee OA.

It is interesting to note that pain in people with knee osteoarthritis may be influenced by involvement of central sensitization at L3, L4, and S2 spinal segments (7). Rehabilitation interventions should target at reducing the secondary spinal sensitization phenomena in case it is present on physical examination. For this reason, it is important to clearly understand the segmental distribution of sensory nerve fibers related to pain originating from the knee joint and periarticular structures. Each segment of the spinal cord and its corresponding spinal nerves have a consistent segmental relationship that allows the clinician to ascertain the probable spinal level of dysfunction based on the pattern of dermatomal, myotomal, and sclerotomal hyperalgesia.

It is important to highlight that nociplastic changes occur not only in the spinal cord, but also in other structures of the central nervous system. Pain due to hip osteoarthritis, for example, is correlated with augmented brain activity at the cingulate cortex, amygdale and thalamus (31). It is very interesting to realize that despite a chronic pain population, we have obtained clinical improvements derived from pain intensity reduction and improvement in the osteoarthritis related functioning status without the need of targeting central cortical structures.

Literature review of current interventions to reduce pain and improve functioning in patients with knee osteoarthritis demonstrate low and very low magnitudes of effect for pain and functioning (32, 33). Estimated number of total knee replacements are exponentially growing and are a burden to the public health systems. Patients keep searching between several services to alleviate the pain. Unmet needs of current treatments contribute to these challenges. Patient-centered treatments based on specific biomarkers as demonstrated in this study acting in the pain roots result in overall well-being related to improvement in pain and function.

According to the current health definition based on “health is not absence of diseases” but physical, social and emotional well-being, a treatment that addresses not only the pain but the weakness, bad habits, malnutrition, sedentarism, poor sleep, and misconceptions. Current guidelines target the symptoms, joint disease, including the anatomical deformities, cartilage damage, but do not address the bone marrow edema (BME) and intraosseous pain perpetuation phenomena. Such interventions are related to low effect size (33). Several authors have already treated BME in patients with osteoarthritis (34–38), this intervention required MRI and local anesthetics.

Patients diagnosed with nociplastic pain present with a decreased responsiveness to peripherally directed therapies such as anti-inflammatory drugs, opioids, surgeries and invasive procedures. First line interventions include non-pharmacological treatments, patient education, promotion of self-management control measures, including proper lifetime habits and psychological therapies. Petersen et al., already reported poor clinical results of 3 weeks of a combination of NSAIDs, analgesics in patients presenting with low pressure pain threshold (PPT) values (11). These authors suggest that low PPTs is an independent predictor for poor pain alleviation by these sets of interventions. On the other hand, our rehabilitation approach targeting the peripheral and segmental sensitization produced pain alleviation and improved functioning in a short period of time. The present report highlights the relevance of proper diagnosis and management of knee OA patients. Patient-centered treatments based on the rationale of augmented or inhibited pain processing based on pain neuroplasticity, work by inhibiting the sensitized pain pathways, and reducing the inhibition of motor pathways (reflex inhibition). It could support the concept that pain centered symptomatic treatments should be replaced by specifically targeted treatment modalities and programs. Isolated surgical or clinical approaches without considering the complex pain system will not be effective. Finally, central changes induced by our rehabilitation protocol seems to be mediated by genetic markers. Compared to non-carriers, participants with polymorphisms on both OPRM1 (A118G) and BDNF (A118G and C17T) seem to have less changes in cortical inhibition (TMS indexed SICI and CSP) (39); thus, underscoring other factors that mediate cortical plasticity.

Our findings also support the idea that besides the arthritis changes in the cartilage surface, properly identified in the RXs, together with proper lack of the joint congruences, the involvement of augmented intraosseous sympathetic input to the dorsal horn of the spinal cord may be involved in the pathogenesis of pain and should be properly identified and addressed (40, 41). Interestingly, these findings also follow a metameric distribution. Radial pressure waves and extracorporeal focal shockwaves therapy have already been used in patients with knee osteoarthritis (42–44). High levels of energy have been already employed for the successful management of bone marrow edema in patients with knee osteoarthritis. The protocol used in previous studies reached up to 0.44 mJ/mm^2^ with high-energy machines (34–36). However, our findings demonstrated that lower doses of 0.12 mJ/mm^2^ have also reduced pain and improved functioning in a large percentage of patients (22).

In this way, our hypothesis is that it is not just an alteration of the surface and joint congruence that causes pain and disability in elderly patients with knee osteoarthritis. There is also an involvement of intraosseous sympathetic innervation related to the compromised metamere. Although we did not use the high dose of focal extracorporeal shockwaves, as employed in other studies (34–36), we still obtained a significant result in pain and with functional improvement in a short time.

On the other hand, ultrasonography is classically recommended to assess joint effusion osteophytes, narrowing of joint space and other features (45). Besides joint effusion, we have also demonstrated a significant percentage of knee OA patients with pes anserine bursitis, patellar tendinitis and popliteal cysts (28). Our study identified USG changes in 78% of the examined knees. Main USG findings were small to moderate joint effusion, pes anserinus bursitis, quadriceps enthesopathy, popliteal cyst, iliotibial band tendonitis and patellar tendinitis. Together with the high prevalence of USG findings, we identified a significant positive correlation between the number of USG findings and pain intensity scores measured by a VAS (28). These periarticular changes are also sources of knee pain and target for interventions including radial shockwave therapies (46–50).

For patients with chronic nonspecific low back pain, the number needed to treat that was associated with the paraspinous block was moderate (approximately 6 in various comparisons) (23). However, because of the safe profile and low cost of this technique, it is an important therapy that should be considered, even for patients with knee osteoarthritis who present with sensitization at the spinal level (23). Paraspinous lidocaine injection does not require the use of imaging techniques to guide its implementation (23). The low operating cost and long-lasting analgesic and functioning benefits, up to 3 months after a 3-week course of treatment encouraged its use in our patients.

Neuromuscular electrical stimulation (NMES) was used to reduce pain and induce muscle contraction. The frequency of 8 Hz with motor level intensity, produces muscle jerks inducing muscle contraction and relaxation without a tetanic fusion of the muscles (24).

Muscle evaluation of aerobic and anaerobic fibers was performed with electrically induced contraction with NMES. The stimulation parameters followed physiological data according to the composition of the muscle fibers. The vastus medialis muscle is composed almost entirely of type I (aerobic) and type IIa (aerobic and anaerobic) fibers, with the proportion of type I fibers being almost twice that of type IIa fibers (51). These data show that the vastus medialis muscle is predominantly aerobic, that is, it is a slow-twitch muscle and more resistant to fatigue. According to muscle physiology studies, the onset frequency for tetanic fusion is different for type I and type II muscle fibers. Type I fibers, of slow contraction, high mitochondrial density, high oxidative capacity and high resistance to fatigue present discharge frequency onset from 7 to 25 Hz, each published work shows a range of variation (25, 26). Type IIb fiber, with rapid muscle contraction, low mitochondrial density, high glycolytic capacity, and low resistance to fatigue, has an onset of discharge frequency from 30 to 65 Hz, and at this frequency would also be stimulating type I fibers as well (25, 26). Based on these studies, we defined that for type I fiber stimulation, a frequency of 20 Hz would be used and for type II fiber work, 60 Hz would be used (which would also stimulate type I).

The vastus medialis muscle has the function of extending the knee, allowing the individual to support their own body weight. For the individual to be able to move from sitting to standing, the quadriceps muscle of each lower limb must be able to support 50% of body weight. Of this percentage approximately 21% represents the strength of the vastus medialis alone which is proportional to the cross-sectional area of the muscle (52). Therefore, as a functional goal of the vastus medialis muscle, 10% of body weight was defined.

After evaluation of the musculature with 20 and 60 Hz, when the musculature did not reach the functional goal of muscle load, treatment was initially performed with 20 Hz until the functional goal and followed with 60 Hz when necessary to reach the functional goal.

We followed the overload principle (53), which consists of a gradual and progressive increase of the external load applied to the limb. To increase size and function, the muscle must be overloaded according to the limit of their response capacity or fatigue (53, 54).

The study's strength is the individualized treatment, centered on patient's individual needs, based on systematized assessments of multiple systems. We have obtained significant improvement in pain and functioning in a short period of time, even for patients with severe osteoarthritis. interventions according to specific findings (biomarkers). We consider it as an appropriate strategy for managing chronic pain conditions in the elderly population, due to its effect, in a short duration, demanding few health professionals and low operational costs. This program should be considered for implementation in middle resourced settings.

Our study has some limitations. Magnetic resonance imaging (MRI) was not used to identify bone or intraosseous changes in our patients. A future study from our research team is already studying the MRI findings in these patients. As an open cohort study, other factors associated with pain improvement are possible, such as a placebo effect. It is also important to highlight that genetic polymorphisms and other biological factors could have influenced overall treatment responses. The present report demonstrated the usefulness of focal extracorporeal shockwaves, radial pressure waves and paraspinous blocks combined with neuromuscular electrical stimulation in the rationale for management of patients with knee OA. The individualized and the multi modal nature for the indication of each intervention according to our decision tree, preclude the possibility to differentiate the effects of the individual treatment modalities.

## Conclusions

The IMREA comprehensive rehabilitation program improved functioning and reduced disabling pain and quality of life in elderly people with knee OA. We highlight the relevance and discuss the implementation of our intervention protocol.

## Data availability statement

The original contributions presented in the study are included in the article, further inquiries can be directed to the corresponding author/s.

## Ethics statement

The studies involving human participants were reviewed and approved by CAAE: 86832518.7.0000.0068. The patients/participants provided their written informed consent to participate in this study.

## Author contributions

LB, MI, and FF contributed equally to conceptualization, investigation, methodology, supervision, and validation. LB and FF further contributed to funding acquisition and managing resources. LB, MI, MS, AdS, and FF contributed to data curation and visualization. AdS conducted formal analysis. LB, MI, MS, GS, SU, AdS, ATS, DM, DA, and TA contributed to writing the original draft, reviewing, and editing. VR assisted with project administration. LB, MI, GS, SU, ATS, DM, and DA all contributed to the investigation. FF provided critical review. All authors contributed to the article and approved the submitted version.

## Funding

This study was supported by a grant from Fundação de Amparo à Pesquisa do Estado de São Paulo (SPEC project, fund number 2017/12943-8). TA was supported by a scholarship from HCFMUSP with funds donated by NUBANK under the #HCCOMVIDA scheme.

## Conflict of interest

The authors declare that the research was conducted in the absence of any commercial or financial relationships that could be construed as a potential conflict of interest.

## Publisher's note

All claims expressed in this article are solely those of the authors and do not necessarily represent those of their affiliated organizations, or those of the publisher, the editors and the reviewers. Any product that may be evaluated in this article, or claim that may be made by its manufacturer, is not guaranteed or endorsed by the publisher.

## References

[1] Hunter DJ Bierma-Zeinstra S. Osteoarthritis. Lancet. (2019) 393:1745–59. 10.1016/S0140-6736(19)30417-9 31034380

[2] Lee JY Han K Park YG Park SH. Effects of education, income, and occupation on prevalence and symptoms of knee osteoarthritis. Sci Rep. (2021) 11:1–8. 10.1038/s41598-021-93394-3 34234235 PMC8263710

[3] Fitzgerald GK Hinman RS Zeni J Risberg MA Snyder-Mackler L Bennell KL. OARSI clinical trials recommendations: design and conduct of clinical trials of rehabilitation interventions for osteoarthritis. Osteoarthr Cartil. (2015) 23:803–14. 10.1016/j.joca.2015.03.013 25952351

[4] Simis M Imamura M Sampaio de Melo P Marduy A Battistella L Fregni F. Deficit of inhibition as a marker of neuroplasticity (DEFINE study) in rehabilitation: a longitudinal cohort study protocol. Front Neurol. (2021) 1193:695406. 10.3389/fneur.2021.695406 34434160 PMC8380986

[5] Dantas LO Salvini T de F McAlindon TE. Knee osteoarthritis: key treatments and implications for physical therapy. Brazilian J Phys Ther. (2021) 25:135–46. 10.1016/j.bjpt.2020.08.004 33262080 PMC7990728

[6] Abbott JH Clare MC McKenzie JE David GD Theis JC Campbell AJ. Exercise therapy, manual therapy, or both, for osteoarthritis of the hip or knee: a factorial randomised controlled trial protocol. Trials. (2009) 10:11. 10.1186/1745-6215-10-11 19200399 PMC2644684

[7] Imamura M Imamura ST Kaziyama HHS Targino RA Wu TH De Souza LPM. Impact of nervous system hyperalgesia on pain, disability, and quality of life in patients with knee osteoarthritis: a controlled analysis. Arthritis Rheum. (2008) 59:1424–31. 10.1002/art.24120 18821657

[8] Roldán-Jiménez C Pérez-Cruzado D Neblett R Gatchel R Cuesta-Vargas A. Corrigendum to: central sensitization in chronic musculoskeletal pain disorders in different populations: a cross-sectional study. Pain Med. (2021) 22:770. 10.1093/pm/pnaa280 32856078

[9] Fitzcharles MA Cohen SP Clauw DJ Littlejohn G Usui C Häuser W. Nociplastic pain: towards an understanding of prevalent pain conditions. Lancet. (2021) 397:2098–110. 10.1016/S0140-6736(21)00392-5 34062144

[10] Treede RD Rief W Barke A Aziz Q Bennett MI Benoliel R. Chronic pain as a symptom or a disease: the IASP classification of chronic pain for the international classification of diseases (ICD-11). Pain. (2019) 160:19–27. 10.1097/j.pain.0000000000001384 30586067

[11] Petersen KK Olesen AE Simonsen O Arendt-Nielsen L. Mechanistic pain profiling as a tool to predict the efficacy of 3-week nonsteroidal anti-inflammatory drugs plus paracetamol in patients with painful knee osteoarthritis. Pain. (2019) 160:486–92. 10.1097/j.pain.0000000000001427 30371559

[12] Altman R Asch E Bloch D Bole G Borenstein D Brandt K. Development of criteria for the classification and reporting of osteoarthritis: classification of osteoarthritis of the knee. Arthritis Rheum Off J Am Coll Rheumatol. (1986) 29:1039–49. 10.1002/art.1780290816 3741515

[13] Bergamasco EC De Cruz DALMD. Adaptation of the visual analog sleep scales to Portuguese. Rev Lat Am Enfermagem. (2007) 15:998–1004. 10.1590/S0104-11692007000500018 18157454

[14] Williamson A Hoggart B. Pain: a review of three commonly used pain rating scales. J Clin Nurs. (2005) 14:798–804. 10.1111/j.1365-2702.2005.01121.x 16000093

[15] Metsavaht L Leporace G Riberto M Sposito MMM Del Castillo LNC Oliveira LP. Translation and cross-cultural adaptation of the lower extremity functional scale into a Brazilian Portuguese version and validation on patients with knee injuries. J Orthop Sports Phys Ther. (2012) 42:932–9. 10.2519/jospt.2012.4101 23047028

[16] Bellamy N Buchanan WW Goldsmith CH Campbell J Stitt LW. Validation study of WOMAC: a health status instrument for measuring clinically important patient relevant outcomes to antirheumatic drug therapy in patients with osteoarthritis of the hip or knee. J Rheumatol. (1988) 15:1833–40. 3068365

[17] Podsiadlo D Richardson S. The timed “Up & Go”: a test of basic functional mobility for frail elderly persons. J Am Geriatr Soc. (1991) 39:142–8. 10.1111/j.1532-5415.1991.tb01616.x 1991946

[18] Steele B. Timed walking tests of exercise capacity in chronic cardiopulmonary illness. J Cardiopulm Rehabil Prev. (1996) 16:25–33. 10.1097/00008483-199601000-00003 8907439

[19] Troosters T Gosselink R Decramer M. Six minute walking distance in healthy elderly subjects. Eur Respir J. (1999) 14:270–4. 10.1034/j.1399-3003.1999.14b06.x 10515400

[20] Berg K. Measuring balance in the elderly: Development and validation of an instrument. Can J Public Health. (1992) 83(Suppl. 2):S7–S11. 1468055

[21] Neder JA Nery LE Shinzato GT Andrade MS Peres C Silva AC. Reference values for concentric knee isokinetic strength and power in nonathletic men and women from 20 to 80 years old. J Orthop Sport Phys Ther. (1999) 29:116–26. 10.2519/jospt.1999.29.2.116 10322586

[22] Cavalieri F Shinzato GT Leite VF Uchiyama SST Miyazaki MM Kirihara AK. Terapia de ondas de choque focal para osteoartrose de joelho: um ensaio clínico randomizado duplo-cego. Acta Fisiátrica. (2017) 24:175–9. 10.11606/issn.2317-0190.v24i4a154184

[23] Imamura M Imamura ST Targino RA Morales-Quezada L Onoda Tomikawa LC Onoda Tomikawa LG. Paraspinous lidocaine injection for chronic nonspecific low back pain: a randomized controlled clinical trial. J Pain. (2016) 17:569–76. 10.1016/j.jpain.2016.01.469 26828801 PMC4910884

[24] Kitchen S Bazin S Bellis E. Electrotherapy: Evidence-Based Practice. London: Churchill Livingstone (2002).

[25] Hannerz J. Discharge properties of motor units in relation to recruitment order in voluntary contraction. Acta Physiol Scand. (1974) 91:374–84. 10.1111/j.1748-1716.1974.tb05692.x 4846331

[26] Kral A BAD T. Functional Electrical Stimulation, Standing and Walking after Spinal Coral Injury. Boca Raton, FL: CRC Press (1989).

[27] Pagano M Gauvreau K. Principles of Biostatistics. Boca Raton, FL: CRC Press (2018).

[28] Abicalaf CARP Nakada LN dos Santos FRA Akiho I dos Santos ACA Imamura M. Ultrasonography findings in knee osteoarthritis: a prospective observational cross-sectional study of 100 patients. Sci Rep. (2021) 11:16589. 10.1038/s41598-021-95419-3 34400659 PMC8367999

[29] Simis M Imamura M Pacheco-Barrios K Marduy A de Melo PS Mendes AJ. EEG theta and beta bands as brain oscillations for different knee osteoarthritis phenotypes according to disease severity. Sci Rep. (2022) 12:1–15. 10.1038/s41598-022-04957-x 35087082 PMC8795380

[30] Simis M Imamura M de Melo PS Marduy A Pacheco-Barrios K Teixeira PEP. Increased motor cortex inhibition as a marker of compensation to chronic pain in knee osteoarthritis. Sci Rep. (2021) 11:24011. 10.1038/s41598-021-03281-0 34907209 PMC8671542

[31] Kulkarni B Bentley DE Elliott R Julyan PJ Boger E Watson A. Arthritic pain is processed in brain areas concerned with emotions and fear. Arthritis Rheum. (2007) 56:1345–54. 10.1002/art.22460 17393440

[32] Arden NK Perry TA Bannuru RR Bruyère O Cooper C Haugen IK. Non-surgical management of knee osteoarthritis: comparison of ESCEO and OARSI 2019 guidelines. Nat Rev Rheumatol. (2021) 17:59–66. 10.1038/s41584-020-00523-9 33116279

[33] Bannuru RR Osani MC Vaysbrot EE Arden NK Bennell K Bierma-Zeinstra SMA. OARSI guidelines for the non-surgical management of knee, hip, and polyarticular osteoarthritis. Osteoarthr Cartil. (2019) 27:1578–89. 10.1016/j.joca.2019.06.011 31278997

[34] Kang S Gao F Han J Mao T Sun W Wang B. Extracorporeal shock wave treatment can normalize painful bone marrow edema in knee osteoarthritis: a comparative historical cohort study. Medicine (Baltimore). (2018) 97:e9796. 10.1097/MD.0000000000009796 29384878 PMC5805450

[35] Gao F Sun W Li Z Guo W Wang W Cheng L. Extracorporeal shock wave therapy in the treatment of primary bone marrow edema syndrome of the knee: a prospective randomised controlled study. BMC Musculoskelet Disord. (2015) 16:379. 10.1186/s12891-015-0837-2 26637992 PMC4670725

[36] Sansone V Maiorano E Pascale V Romeo P. Bone marrow lesions of the knee: longitudinal correlation between lesion size changes and pain before and after conservative treatment by extracorporeal shockwave therapy. Eur J Phys Rehabil Med. (2019) 55:225–30. 10.23736/S1973-9087.18.05036-0 30156085

[37] Vitali M Naim Rodriguez N Pedretti A Drossinos A Pironti P Di Carlo G. Bone marrow edema syndrome of the medial femoral condyle treated with extracorporeal shock wave therapy: a clinical and MRI retrospective comparative study. Arch Phys Med Rehabil. (2018) 99:873–9. 10.1016/j.apmr.2017.10.025 29223709

[38] Häußer J Wieber J Catalá-Lehnen P. The use of extracorporeal shock wave therapy for the treatment of bone marrow oedema - a systematic review and meta-analysis. J Orthop Surg Res. (2021) 16:369. 10.1186/s13018-021-02484-5 34107978 PMC8188716

[39] de Toledo Gonçalves F Pacheco-Barrios K Rebello-Sanchez I Castelo-Branco L de Melo PS Parente J. Association of Mu opioid receptor (A118G) and BDNF (G196A) polymorphisms with rehabilitation-induced cortical inhibition and analgesic response in chronic osteoarthritis pain. Int J Clin Heal Psychol. (2023) 23:100330. 10.1016/j.ijchp.2022.100330 36199368 PMC9508345

[40] Zhang X Hassan MG Scheller EL. Neural regulation of bone marrow adipose tissue. Best Pract Res Clin Endocrinol Metab. (2021) 35:101522. 10.1016/j.beem.2021.101522 33766429 PMC8440660

[41] Maryanovich M Takeishi S Frenette PS. Neural regulation of bone and bone marrow. Cold Spring Harb Perspect Med. (2018) 8:a031344. 10.1101/cshperspect.a031344 29500307 PMC6119651

[42] Hsieh C-K Chang C-J Liu Z-W Tai T-W. Extracorporeal shockwave therapy for the treatment of knee osteoarthritis: a meta-analysis. Int Orthop. (2020) 44:877–84. 10.1007/s00264-020-04489-x 31993710

[43] Wang Y-C Huang H-T Huang P-J Liu Z-M Shih C-L. Efficacy and safety of extracorporeal shockwave therapy for treatment of knee osteoarthritis: a systematic review and meta-analysis. Pain Med. (2020) 21:822–35. 10.1093/pm/pnz262 31626282

[44] Avendaño-Coy J Comino-Suárez N Grande-Muñoz J Avendaño-López C Gómez-Soriano J. Extracorporeal shockwave therapy improves pain and function in subjects with knee osteoarthritis: a systematic review and meta-analysis of randomized clinical trials. Int J Surg. (2020) 82:64–75. 10.1016/j.ijsu.2020.07.055 32798759

[45] Katz JN Arant KR Loeser RF. Diagnosis and treatment of hip and knee osteoarthritis: a review. JAMA. (2021) 325:568–78. 10.1001/jama.2020.22171 33560326 PMC8225295

[46] Zhao Z Jing R Shi Z Zhao B Ai Q Xing G. Efficacy of extracorporeal shockwave therapy for knee osteoarthritis: a randomized controlled trial. J Surg Res. (2013) 185:661–6. 10.1016/j.jss.2013.07.004 23953895

[47] Wei M Liu Y Li Z Wang Z. Comparison of clinical efficacy among endoscopy-assisted radio-frequency ablation, extracorporeal shockwaves, and eccentric exercises in treatment of insertional achilles tendinosis. J Am Podiatr Med Assoc. (2017) 107:11–6. 10.7547/14-146 27723374

[48] Xu Y Wu K Liu Y Geng H Zhang H Liu S. The effect of extracorporeal shock wave therapy on the treatment of moderate to severe knee osteoarthritis and cartilage lesion. Medicine (Baltimore). (2019) 98:15523. 10.1097/MD.0000000000015523 31096453 PMC6531190

[49] Hammam RF Kamel RM Draz AH Azzam AA Abu El Kasem ST. Comparison of the effects between low- versus medium-energy radial extracorporeal shock wave therapy on knee osteoarthritis: a randomised controlled trial. J Taibah Univ Med Sci. (2020) 15:190. 10.1016/j.jtumed.2020.04.003 32647513 PMC7336004

[50] Zhang YF Liu Y Chou SW Weng H. Dose-related effects of radial extracorporeal shock wave therapy for knee osteoarthritis: a randomized controlled trial. J Rehabil Med. (2021) 53:jrm00144. 10.2340/16501977-2782 33367924 PMC8772366

[51] Travnik L Djordjevič S Rozman S Hribernik M Dahmane R. Retracted: muscles within muscles: a tensiomyographic and histochemical analysis of the normal human vastus medialis longus and vastus medialis obliquus muscles. J Anat. (2013) 222:580–7. 10.1111/joa.12045 23586984 PMC3666237

[52] O'Brien TD Reeves ND Baltzopoulos V Jones DA Maganaris CN. Muscle–tendon structure and dimensions in adults and children. J Anat. (2010) 216:631–42. 10.1111/j.1469-7580.2010.01218.x 20345856 PMC2871999

[53] De Lorme TL. Heavy resistance exercises. Arch Phys Med Rehabil. (1946) 27:607–30. 21000039

[54] Enoka RM. Neuromechanics of Human Movement. Human kinetics; (2008).

